# Three Cases of Organized Hematoma of the Maxillary Sinus: Clinical Features and Immunohistological Studies for Vascular Endothelial Growth Factor and Vascular Endothelial Growth Factor Receptor 2 Expressions

**DOI:** 10.1155/2015/846832

**Published:** 2015-01-29

**Authors:** Shoichiro Imayoshi, Takeharu Kanazawa, Noriyoshi Fukushima, Hisashi Kikuchi, Masayo Hasegawa, Takafumi Nagatomo, Hiroshi Nishino

**Affiliations:** ^1^Department of Otolaryngology, Head and Neck Surgery, Jichi Medical University School of Medicine, 3311-1 Yakushiji, Shimotsuke, Tochigi 329-0498, Japan; ^2^Department of Pathology, Jichi Medical University School of Medicine, 3311-1 Yakushiji, Shimotsuke, Tochigi 329-0498, Japan

## Abstract

*Objectives*. Organized hematoma (OH) is a rare, nonneoplastic, hemorrhagic lesion causing mucosal swelling and bone thinning, mainly in the maxillary sinus. We aimed to clarify the clinical presentation and treatment of OH. *Methods*. Three cases of maxillary sinus OH and a literature review are presented. *Results*. Three men aged 16–40 years complained of nasal obstruction, frequent epistaxis, and/or headache. Clinical and radiological examinations revealed a maxillary sinus OH. They were cured in a piecemeal fashion via endoscopic middle meatal antrostomy. Furthermore, vascular endothelial growth factor and its receptor were expressed in the lesion. *Conclusions*. The pathogenesis of OH is unclear and it presents various histological and imaging findings; however, it is not difficult to rule out malignant tumors. Minimally invasive surgery such as endoscopic sinus surgery can cure it completely. Thus, it is important to determine the diagnosis using CT and MRI and to quickly provide surgical treatment.

## 1. Background

An organized hematoma (OH) is rare, mainly arises from the maxillary sinus, and is a nonneoplastic, hemorrhagic lesion causing mucosal swelling and bone thinning [1]. The first report of this lesion was introduced in the Japanese literature as a “blood boil” by Tadokoro in 1917, and it is relatively difficult to differentiate it from a malignant lesion causing mucosal swelling and bone thinning [2, 3]. Thus far, many reports have described the imaging findings and/or clinical course, but the pathogenesis of the disease is unclear. Relatively invasive surgery, such as transmaxillary approach and lateral rhinotomy, was described in previous reports [4, 5]. Recently, reports of treatment by endoscopic sinus surgery are increasing [2, 6]. In this case series, we report three cases of OH that were cured by a piecemeal approach via an endoscopic middle meatal antrostomy. Furthermore, we first demonstrated that vascular endothelial growth factor (VEGF) receptor 2 (VEGFR2) is expressed on the vascular endothelial cells of dilated vessels. VEGF is also expressed in inflammatory cells around dilated vessels. These expressions could be related to the pathogenesis of OH.

## 2. Case Presentation

Three patients with OH of the maxillary sinus who were treated in our department from 2010 to 2011 were retrospectively analyzed on the basis of their medical records. The patients' clinical features are summarized in Table 1.

### 2.1. Case 1

**Figure 1:**
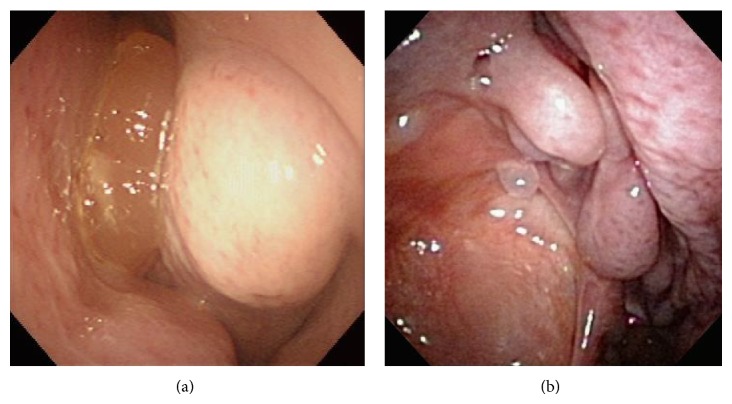
On paranasal endoscopy, an extension of the medial wall of the right maxillary sinus to the nasal side with a polyp is observed in Case 1 (a). Postoperatively, the maxillary sinuses are well-opened, and no evidence of recurrence is observed (b).

**Figure 2:**
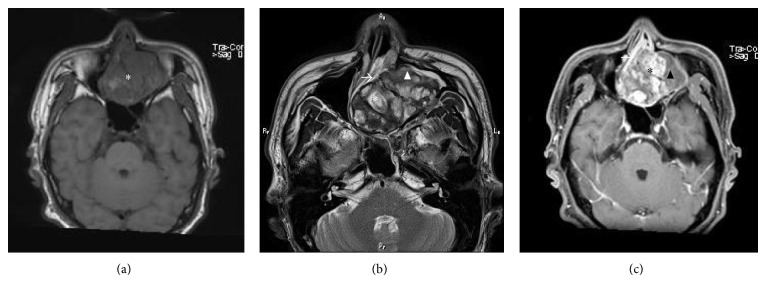
MRI findings of Case 1. (a) On T_1_-weighted imaging, the central region (∗) has a slightly lower intensity compared to that of the muscle. (b) On T_2_-weighted imaging, the central region (∗) demonstrates high intensity, and the periphery (▲) has lower intensity than the central region. Thickening of the paranasal mucosa is observed (→). (c) On contrast-enhanced T_1_-weighted imaging, the central region is strongly enhancing (∗), while the periphery (▲) demonstrates less enhancement than the central region. Additionally, the thickened mucosa of the paranasal sinus are strongly enhanced (→).

A 35-year-old man had frequent epistaxis and nasal obstruction. None had a past history of paranasal sinus surgery or trauma or was receiving anticoagulant drugs for the treatment of an underlying disease. On paranasal endoscopy, an extension of the medial wall of the right maxillary sinus to the nasal side with a polyp and (Figure 1(a)) a blood clot was observed. CT showed an expansile, homologous, enhancing 7 × 5 cm sized mass with bone destruction of the maxillary sinus. On MRI, low-to-iso-intensity was observed in the periphery on T_1_-weighted imaging (Figure 2(a)) and iso-to-high intensity was observed on T_2_-weighted imaging (Figure 2(b)). The central region was more strongly enhancing than the periphery (Figure 2(c)). The surrounding paranasal sinus mucosa was thickened. However, the central region showed low intensity and was strongly enhanced by gadolinium on T_1_-weighted imaging and demonstrated high intensity on T_2_-weighted imaging. Pathologic evaluations were performed, but there was no evidence of malignancy. The lesions were curetted by a piecemeal approach via an endoscopic middle and inferior meatal antrostomy with the assistance of a microdebrider. The masses were brittle and hemorrhagic, but it was easy to exfoliate the capsule from the maxillary mucosa. Complete removal of the tumor was accomplished, and the remainder of the maxillary sinus mucosa was slightly edematous and was kept intact. The total blood loss during surgery was 700 mL, and no blood transfusion was needed. Operation time was 127 min. The maxillary sinuses were well-opened and no evidence of recurrence was observed (Figure 1(b)). There were no postoperative complications, and the postoperative courses of the patients were uneventful.

### 2.2. Case 2

A 16-year-old man had nasal obstruction and headache without significant past history and anticoagulant drug use. Paranasal endoscopic examination revealed an extension of the medial wall of the right maxillary sinus to the nasal side with a polyp. CT showed an expansile, homologous, enhancing mass with bone destruction of the maxillary sinus. The size of the mass was 5 × 5 cm. MRI images were similar to Case 1. Briefly, low intensity was observed in the periphery on T_1_-weighted imaging and iso intensity was observed on T_2_-weighted imaging. The central region showed low intensity and was strongly enhanced by gadolinium on T_1_-weighted imaging and demonstrated high intensity on T_2_-weighted imaging. Pathologic evaluations indicated no evidence of malignancy. The lesions were curetted by a piecemeal approach via an endoscopic middle meatal antrostomy with the assistance of a microdebrider. Complete removal of the tumor was accomplished. The total blood loss was 300 mL, and no blood transfusion was needed. Operation time was durations 95 min. The maxillary sinuses were well-opened and no evidence of recurrence was observed.

### 2.3. Case 3

A 40-year-old man had frequent epistaxis, and none had a significant past history or was receiving anticoagulant drugs. Paranasal endoscopic examination revealed an extension of the medial wall of the right maxillary sinus to the nasal side with a polyp. CT and MRI images supported the specific features of OH, the same as in Cases 1 and 2. The size of mass was 6 × 4 cm. Pathologic evaluation indicated no evidence of malignancy. Complete removal of the tumor was accomplished by an endoscopic middle meatal antrostomy. The total blood loss during surgery was 450 mL, and no blood transfusion was needed. Operation time was from 155 min. There were no postoperative complications, and the postoperative courses of the patients were uneventful.

**Figure 3:**
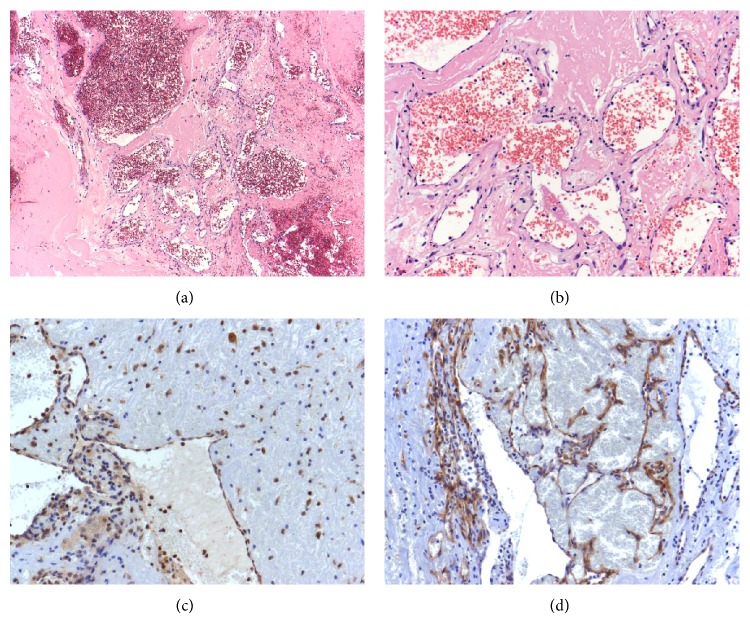
The lesion consisted of dilated vessels, hemorrhage, fibrin, exudation, and fibrosis with hyalinization. Atypical cells are not seen (hematoxylin and eosin staining; (a) original magnification, ×40; (b) original magnification, ×200). VEGF is expressed in endothelial and inflammatory cells around the dilated vessels ((c) original magnification, ×200). VEGFR2 is expressed on the vascular endothelial cells of the dilated vessels ((d) original magnification, ×400).

Histologically, the specimens of all cases consisted of dilated vessels, hemorrhage, fibrin, exudation, fibrosis, hyalinization, and neovascularization without atypical cells (Figures 3(a) and 3(b)). Furthermore, to help elucidate the pathogenesis of OH, we checked the expressions of VEGF and VEGFR by immunohistochemistry. The antibodies for VEGF (Santa Cruz Biotechnology, Dallas, TX, USA) and VEGFR2 (Cell Signaling Technology, Beverly, MA, USA) used concentrations of 1 : 100 and 1 : 200. In all three cases, VEGF was expressed in endothelial and inflammatory cells such as neutrophils, macrophages, and plasma cells around the dilated vessels (Figure 3(c)). VEGFR was expressed on the vascular endothelial cells of the dilated vessels (Figure 3(d)).

## 3. Discussion

The term “organized hematoma” is not unified in the English literature and many terms, such as blood boil, hematoma-like mass, hematocele, blutbeule, and organizing hematoma, are used [1]. Song et al. stated that “organization” refers to “replacement of hematoma by fibrous tissue” and that the recently proposed term “hematoma-like mass” is inadequate to explain its histopathological and clinical behavior [1, 3]. Recently, the number of reports about the clinical characteristics has been increasing, but the pathogenesis is not fully understood [1–7]. Common symptoms related to OH include frequent epistaxis, nasal obstruction, cheek swelling, and exophthalmos [8]. In our series, frequent epistaxis, nasal obstruction, and headache were observed most commonly, and patients desired relief from these severe symptoms. OH typically occurs in patients from 20 to 40 years of age, as in our reports, and the age of patients at diagnosis is younger than that of maxillary carcinoma [1].

A correct preoperative diagnosis is important for determining a therapeutic plan, and CT and MRI are strongly recommended [1, 3, 6–8]. The reported CT appearances of sinonasal OHs are rather nonspecific and typically include a large mass causing expansion of the maxillary sinus with bony erosion; patchy, heterogeneous enhancement to various degrees is usually found within the lesion on contrast-enhanced CT [1, 6, 8–10]. MRI shows thickening of the surrounding paranasal sinus mucosa. The mucosa are well-enhanced on T_1_-weighted imaging with contrast, and high intensities are observed on T_2_-weighted imaging. It is suggested that inflammatory changes result from obstruction by the lesion. These points differ from a malignant tumor that causes invasive bone destruction and from mucosal invasion. The existence of blood with low flow was thus considered in the central region, which matched the presence of a hematoma in the pathological findings. However, the peripheral regions were less enhanced and matched the zone of fibrosis. Therefore, a biphasic appearance is an important imaging characteristic of this lesion and it is important to cite in the differential diagnosis before determining a treatment strategy [1]. Hur et al. concluded that sinonasal organized hematoma can be mistaken for a malignant tumor; the characteristic imaging findings such as erosion of the bony sinus walls, markedly heterogeneous signal intensity, and papillary or frond-like enhancement facilitate the diagnosis of an OH [10].

The pathogenesis of maxillary OH is not fully understood. A possible mechanism for the formation of OH has been suggested [1]. According to this theory, (1) repeated hemorrhage in a semiclosed lumen (maxillary sinus) forms a hematoma that is encapsulated by fibrosis; (2) thus, this increases pressure within the hematoma; and (3) progressive expansion of the hematoma causes demineralization of adjacent structures. Namely, OH might represent aseptic inflammation-induced bleeding with a hematoma in an enclosed space. Ohta et al. demonstrated CD31 and CD34 expression in this type of lesion to examine the negative spiral theory [9]. The results showed that the endothelial cells kept their structural integrity and dilated pattern in a limited area. CD34 expression indicated activated neovascularization observed in the lesion [9]. Yokoi et al. also found primary angiomatous lesions and the presence of proliferative activity [6]. VEGF is a signal protein produced by cells that stimulates vasculogenesis and angiogenesis. We observed that the lesions that highly expressed VEGF/VEGFR2 have high degree of vascularization and that the other inflammatory lesions of extra-OH in the same specimens have less expression of VEGF/VEGFR2. It has been shown that VEGF and its receptors were related to the neovascularization of OH.

We are the first to demonstrate that VEGF is expressed in nearby inflammatory cells and VEGFR is expressed in parts of the hemangioma. Although statistical analysis could not be performed because of being short of cases, these data provide some evidence that VEGF and VEGFR2 expression are related to the cause of OH.

Surgery is mandatory to remove the OH as first-line treatment strategy. Preoperative embolization can decrease the intraoperative bleeding volume but is unnecessary in all cases [1, 2]. Previous cases have undergone relatively invasive surgeries such as a Caldwell-Luc operation, Denker's operation, or a lateral rhinotomy approach [4, 5]. Recently, removal by an endoscopic method, a less invasive surgical technique, is the most common surgical approach [2, 6]. The reasons for this change might result from the development of endoscopic surgery and an understanding that the pathogenesis of OH is aseptic, inflammation-induced bleeding with a hematoma in an enclosed space, not a neoplasm. Our experience indicated that complete removal was possible from the enlarged middle nasal meatus because it is easy to exfoliate the capsule from the maxillary mucosa. Actually, all cases in this series were removed completely by a middle meatal antrostomy and did not require combination with a transmaxillary approach. On the other hand, Suzuki et al. described that complete removal is unnecessary to cure the OH, because even if a small amount of remaining tissue was left in the sinus, the disease was eventually cured in all their cases [2]. Presently, endoscopic surgery is becoming the main treatment approach for OH.

As a matter of course, the important things are to verify the surgical method to other approaches when ESS is not easy for OH removal during the surgery and to discuss with patients the possibility of modification of surgical approach during the surgery preoperatively.

## 4. Conclusion

The pathogenesis of OH is unclear, and OH presents various findings in histological and imaging diagnoses, but it is not difficult to rule out malignant tumors. Furthermore, minimally invasive surgeries such as endoscopic sinus surgery can cure it completely. Thus, it is important to determine the diagnosis using CT and MRI and to treat the lesion quickly with a surgical operation.

## Figures and Tables

**Table 1:** Clinical characteristics of the three cases.

Case	Age (y)/sex	Chief complaint	Side	Size (cm)	Surgical approach	Total blood loss (mL)	Operation time (min)
1	35M	Frequent epistaxis Nasal obstruction	L	7 × 5	Middle and inferior meatal antrostomy	700	127
2	16M	Nasal obstruction Headache	R	5 × 5	Middle meatal antrostomy	300	95
3	40M	Frequent epistaxis	R	6 × 4	Middle meatal antrostomy	450	155

## Abbreviations

OH: Organized hematoma
VEGF: Vascular endothelial growth factor
VEGFR2: Vascular endothelial growth factor receptor 2
CT: Computed tomography
MRI: Magnetic resonance imaging.
